# Blood-letting in Symptomatic Fevers

**Published:** 1866-04

**Authors:** S. R. Millard

**Affiliations:** Aurora, Ill.


ARTICLE XIV.

### BLOOD-LETTING IN SYMPTOMATIC FEVERS.

By S. R. MILLARD, M.D., Aurora, Ill.

Read to the Aurora Medical Society.

By the term symptomatic fever, I mean, of course, fever depending for its origin on some acute inflammation.

Let us now look at this matter of substitution. In the first place, there is no such thing in nature, strictly speaking, as substitution. All things have their legitimate spheres and specific influences. The idea of exact substitution involves the absurdity of the existence of some things which can, without loss or injury, be dispensed with—an unnecessary creation. Wherever one thing absolutely supplies the place of another, it will be found that these seemingly different things are really identical.

To examine properly the nature and powers of substitutes for blood-letting in acute inflammations, let us consider common pneumonia, with or withour pleuritis, and as a sample of the characteristics of inflammation and of the influence of blood-letting upon it. I do not propose to describe the disease, nor its treatment, in detail. I shall speak only to the points I raise. I choose pneumonia, because it is the disease in which veratrum viride is supposed to be particularly efficacious as a substitute for venesection. In all acute inflammatory affections, the powers of the system are elevated, as a general principle, above the natural standard. The exception is, when some vital part, as the delicate and highly complicated mucous membrane, is inflamed, and then the disease has a tendency to depression; on the principle of an extensive injury to the fibrils of the nervous system. But I will not dwell upon exceptions. In pneumonia—an affection of parenchymatous structure—this injurious shock does not obtain; the powers and resiliency of the system are elevated; there is active congestion of a large portion of the lung. This is necessarily attended by a withdrawal of that portion of the lung from its duty in assisting the proper aëration of the blood, so that, while in health, the entire lungs are called into action to perform the function of respiration upon the blood in its natural circulation, and in a healthy condition. In pneumonia, a portion of the lung is quiescent and useless, while the circulation is greatly increased. This produces head symptoms, and, in addition, a poisonous state of the blood, such as acts upon the nervous sensibilities in a way inimical to the healthy action of remedies, not to mention the proper action of the secretory functions.

What does blood-letting effect in a condition of this kind? I will look at its effects *seriatim*, and we will see how it is that any internal remedy cannot possibly occupy the position of a "substitute for blood-letting:—"

1st. The abstraction of blood restores the equilibrium between the amount of blood in circulation and the air respired, which had been impaired by the inactivity of a portion of the lungs, by reducing the amount of the circulating medium. It also relieves by restoring the just relationship between the circulation and respiration, the oppression of the nervous system, depending upon unaërated or badly aërated blood circulating in the arterial capillaries. In this way it awakens the natural sensibilities of the system, thus promoting the effects of other remedies.

Will veratrum or digitalis as effectually restore this equilibrium? Veratrum is powerless in fulfilling this important indication—an indication that should never be overlooked in any serious case of pneumonia. I will not dwell upon the bad complications that are liable to ensue if it is neglected.

2d. Does not every physician know that venesection is infinitely the most powerful measure that can be employed in promoting absorption?

I have heard it contended that pneumonia is to be got rid of by expectoration. Loosening the cough is not intended to enable the patient to spit up the disease; it matters little whether veratrum renders expectoration easy or not, if it will do no more as a substitute for bleeding in the disease under consideration. Proper venesection will also loosen the cough; but instead of making expectoration copious, it diminishes its quantity. It does this by so emptying the bloodvessels and relieving the oppressed powers of the nervous system, that the accumulated matter (the blood and lymph congesting the lungs) reënters the circulation, back again, the same way it came out of it; and thus the oppression of the lungs is relieved, the necessity of copious expectoration fades away, and the affected part speedily begins to resume, if proper adjuvant treatment is employed, its wonted functions.

Will veratrum thus fill the place of blood-letting in removing the inflammation and congestion of pneumonia? The pretension is perfectly inconsistent.

3d. In symptomatic fevers, bleeding promotes the operation of medicines. Critical discharges are more readily and freely brought about, either in the form of evacuations from the bowels, or of the secretions of the kidneys or skin, subsequently to venesection, than they are before it has been resorted to. This especially obtains in pneumonia, in consequence of the absorption of medicines being promoted by the remedy, and because the nervous system is not merely relieved temporarily from the influence of fever, but because, also, it is permanently relieved from the poisonous oppression of unaërated blood—a relief that, in the nature of things, veratrum is inadequate so fully to afford.

I am not disposed to deny to veratrum certain useful properties in the treatment of inflammation; but that it is ever a substitute for blood-letting, I most emphatically deny. In severe cases of pneumonia we have more blood than we want. It is in our way, and in the way of convalescence. We want to get rid of it, and if there is any boon we should thank Providence for, in such circumstances, it is the knowledge and experience which directs blood-letting.

